# Non-Cariogenic Effect of Milk and Dairy Products on Oral Health in Children and Adolescents: A Scoping Review

**DOI:** 10.3390/children11020149

**Published:** 2024-01-24

**Authors:** Flavia Vitiello, Denis Bourgeois, Giulia Orilisi, Giovanna Orsini, Florence Carrouel

**Affiliations:** 1 Laboratory “Health, Systemic, Process” (P2S), UR4129, University Claude Bernard Lyon 1, University of Lyon, 69008 Lyon, France; f.vitiello@pm.univpm.it (F.V.); denis.bourgeois@univ-lyon1.fr (D.B.); 2 Department of Clinical Sciences and Stomatology (DISCO), Università Politecnica delle Marche, 60126 Ancona, Italy; g.orilisi@pm.univpm.it (G.O.);

**Keywords:** dental caries, milk, dairy products, tooth demineralization, cariogenic agents

## Abstract

Diet plays an important role in the etiopathology of dental caries. Milk and dairy products (DPs), especially in children and adolescents, are important sources of protein, calcium and phosphorus and could have an effect on dental and oral health. The aim of this scoping review was to analyze the scientific literature on the non-cariogenic effect of milk and DPs, with a focus on their potential to prevent dental caries in children and adolescents. PubMed, EMBASE, and Web of Science were searched for publications relevant to our topic from January 2013 to 30 September 2023. Thirty-eight studies were included in the qualitative analysis. The included studies highlight the properties of milk and DPs that contribute to enamel remineralization, exhibit antibacterial action, inhibit the growth of cariogenic bacteria, and promote a balanced oral microbiome. With regard to the addition of probiotics (PBs) and fluoride (F) to DPs, the mixed results of the studies analyzed did not allow a clear statement to be made about their non-cariogenic effects. However, several studies show that the addition of PBs can reduce cariogenic bacteria, create a protective barrier against pathogens and support the host’s natural defense mechanisms. Further long-term and high-quality studies are needed to understand the impact of milk and its constituents on oral health in order to promote effective caries prevention strategies in children and adolescents.

## 1. Introduction

Dental caries remains a significant global health burden, affecting an estimated 2.5 billion people worldwide [1]. In primary teeth, untreated caries stands out as the most common chronic disease of childhood, affecting 514 million children, as documented in the World Health Organization (WHO) report on Global Oral Health Status (2022) [2]. 

According to the American Academy of Pediatric Dentistry (AAPD), dental caries is a biofilm-induced, saliva-mediated acid demineralization of enamel or dentin [3]. It is a common, complex, chronic disease resulting from a complex interaction between the microbiota, host susceptibility, environmental factors and diet [4]. The pathological process starts in the biofilm, where bacteria metabolize carbohydrates to organic acids, lowering the pH levels, and causing the demineralization of dental tissues [5]. Prolonged dysbiosis of the microbiota can progress with an increase in acid-forming species, such as *Streptococcus mutans* (*S. mutans*), which is the leading cause of dental caries in humans [6], and *Lactobacillus*, which continues to produce acids, further damaging dental hard tissues [7]. Saliva has a buffering system that neutralizes acids produced by acidogenic microorganisms through the action of inorganic ions, such as calcium, phosphate, hydroxyl, and fluoride (F), thus preventing enamel demineralization [8].

Although the role of bacteria in the development of a carious lesion is crucial, dental caries is considered a multifactorial disease [9], leading researchers to explore the intricate relationship between dietary habits and oral health [10,11]. In this field, milk and dairy products (DPs) are considered an essential part of the human diet, especially in children and adolescents, as they not only provide sufficient nutrients and energy, but also have beneficial effects on dental and oral health, including hard tissue remineralization, salivary flow stimulation, oral microbiota and immune response regulation, thus directly or indirectly influencing caries susceptibility [12]. In particular, milk is considered to be a rich source of essential nutrients such as calcium and phosphorus, which promote overall general health [13]. In fact, adequate calcium intake during childhood and adolescence is crucial for achieving optimal peak bone mass, including dental mineralization [14]. DPs are derived from mammalian milk and include milk itself, cream, butter, cheese, yogurt, frozen desserts and whey. They contain a variety of micro- and macronutrients, namely saturated fatty acids [15]. These foods are also excellent sources of protein, in particular casein, which is a major protein in milk and has a protective effect on dental enamel by forming a protective film on the tooth surface [16]. This film may help to reduce susceptibility to acid erosion and caries [17,18].

In addition, some DPs contain lactose, a natural sugar that stimulates the production of saliva, which helps to neutralize the acidity of the oral microenvironment. Conversely, some sweetened DPs may contain added sugars that can contribute to the development of caries. It is, therefore, important to distinguish between the cariogenic potential of the sugars in these products and the non-cariogenic properties of milk constituents [19].

In addition to their intrinsic potential, milk and DPs can be combined with other supplements such as probiotics (PBs) or F to enhance their anticariogenic effect [20,21]. The use of PBs helps to maintain the oral ecological balance, by regulating the pathogenicity of biofilm, and restoring the healthy microbiota [22,23]. In fact, they compete with the cariogenic bacteria such as *S. mutans* for the carbohydrate substrate, thereby reducing their numbers [24]. 

This complex dichotomy of cariogenic and non-cariogenic factors in milk and DPs is a challenge for researchers and healthcare professionals. In order to meet this challenge, it is imperative to have in-depth knowledge on the molecular and microbial interactions that occur in the oral cavity following the consumption of DPs.

The consumption of DPs by children and adolescents is a critical aspect of oral health and overall nutritional well-being [25]. During the growth and development of primary and permanent teeth, it is essential to maintain strong and healthy teeth; therefore, it is important to evaluate the impact of DPs consumption on the risk of dental caries in this specific age group. However, there is still a lack of relevant research and confounding data on this topic. Therefore, the aim of this study was to summarize the existing updated landscape to better understand the non-cariogenic effect of milk and DPs on dental caries in children and adolescents by conducting a scoping review of articles from the last decade to describe current evidence and findings.

## 2. Materials and Methods

### 2.1. Research Question

This review is based on the Preferred Reporting Items for Systematic reviews and Meta-Analyses extension for Scoping Reviews (PRISMA-ScR) checklist [26] (Supplementary Table S1). The PICO research question was “Do milk and dairy products (I) have a non-cariogenic effect (O) in children and adolescents (P) compared with other substances, treatments or placebo (C)?”

### 2.2. Search Strategy

An electronic literature search was conducted by two examiners (FV and FC) in the databases PubMed, EMBASE and Web of Science. The searches were performed on 30 September 2023 and included studies published from 2013 to 2023. For Medline/PubMed, search strategies using MeSH and free terms combined with Boolean operators (OR, AND) were developed and adapted to the syntax rules of each database. The search terms were “dairy products” OR “probiotics” OR “milk” OR “yogurt” OR “cheese” OR “lactose” OR “casein” AND “caries” OR “enamel demineralization” OR “decay” OR “cavities” OR “cavity” OR “carious” AND “tooth” OR “teeth” AND “children” OR “child” OR “adolescent”. After merging the results of the three databases, duplicates were eliminated.

### 2.3. Screening and Eligibility Criteria

Articles were included if they were (i) published between 2013 and 2023; (ii) written in English; (iii) fully accessible without restriction; (iv) focused on the cariogenic or anticariogenic effect of dairy products; (v) focused on children and adolescents. Articles were excluded if they were (i) case reports/case series, posters, commentaries, opinions, letters, editorials, conference abstracts or (ii) in vitro studies not related to the scope of this review.

The eligibility of articles was checked by analyzing titles and abstracts. Screening and selection of publications was performed independently by two investigators (FV and FC) using predefined inclusion and exclusion criteria. For studies that appeared to meet the inclusion criteria, the full text was obtained and reviewed. In case of disagreement, the two reviewers discussed each step until consensus was reached.

### 2.4. Data Extraction and Quality Assessment 

Data extraction was performed by one investigator (FV) and checked by a second (FC). For each included study, the main data were extracted: citation details, study design, population, objectives, intervention, duration and follow-up, results, and conclusions. The quality of the included studies was assessed by the two reviewers (FV and FC) using the National Institutes of Health’s study quality assessment tools [27]. In case of disagreement, the two reviewers conferred until a consensus was reached. 

### 2.5. Search Outcome and Evaluation

The objective of this scoping review was to assess whether there is clear evidence of beneficial effects of milk and DP consumption on oral health, with a focus on non-cariogenic aspects in children and adolescents. To this end, the existing literature was mapped by classifying the types of dairy supplementation into milk and DPs, milk and DPs supplemented with PBs, and milk and DPs supplemented with F.

## 3. Results

### 3.1. Selection of Publications Included

**Figure 1:**
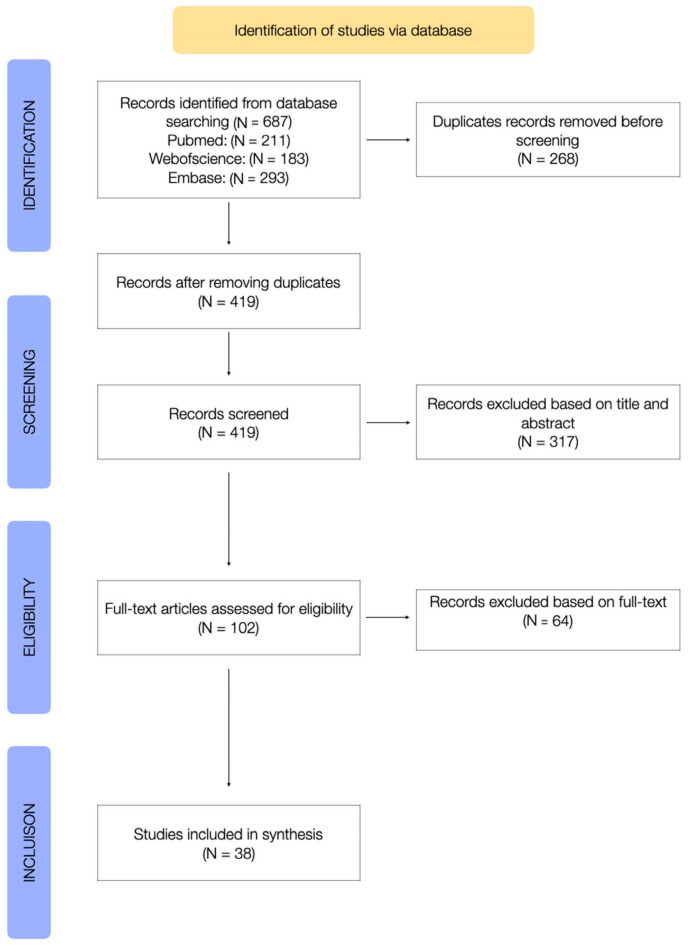
Flowchart of study and selection process.

Figure 1 shows the PRISMA-ScR study flowchart describing the different steps of article selection. From the initial database search, 687 papers were identified. After removing duplicates, 419 papers were screened at title and abstract level, and 102 potentially relevant full text articles were selected for eligibility assessment. Finally, 38 publications met the inclusion criteria and were included in the review.

### 3.2. Characteristics of Publications Included

The main results of the included studies are summarized in Table 1, Table 2 and Table 3. Among the 38 included studies, there were 16 randomized controlled trials (RCT), two case–control studies, two pre-post studies, 10 observational studies, and eight systematic reviews and meta-analyses.

### 3.3. Synthesis of the Results

The results of the analysis were summarized and classified according to the products studied.

#### 3.3.1. Milk and DPs

Thirteen articles analyzed the effect of milk and DPs on caries (Table 1). Sukmana et al. studied the relationship between breastfeeding and the occurrence of early childhood caries (ECC), lesions present in the primary dentition of a child under 6 years of age, as defined by AAPD, and concluded that the risk of ECC in breastfeeding children may increase after the first intake of complementary foods [28]. On the same topic, Neves et al. showed that breastfeeding did not cause a significant decrease in biofilm pH in caries-free or ECC children. Thus, this study suggests that breastfeeding may not contribute to the formation of ECC [29]. Regarding the duration of breastfeeding, Branger et al. found that breastfeeding is a protective factor for ECC under 1 year of age and is not associated with an increased risk of dental caries [30]. Consistent with this, a prospective study conducted in a population at high risk of caries suggested the benefit of exclusive breastfeeding for 6–11 months for the prevention of dental caries in primary teeth [31]. Beyond 1 year, there may be confounding factors such as dietary patterns that could influence the assessment [30].

Wang et al. investigated the associations between milk (i.e., whole milk, low-fat milk, skim milk and total fluid milk) and DPs (i.e., yogurt, cheese, cream and dairy desserts) and the risk of dental caries in American children and adolescents, suggesting that high consumption of yogurt and low consumption of cheese were associated with lower risk of dental caries [32]. Similar results were shown by Zaki et al., who demonstrated that milk and cheese reduce the effects of metabolic acids by reducing the acidity of plaque and consequently its cariogenicity [33]. 

Hegde et al. compared the salivary levels of calcium, phosphate and alkaline phosphatase levels in children with ECC after ingestion of milk, cheese and casein phosphopeptides and amorphous phosphate (CPP-ACP) mousse with a control group of caries-resistant children [34]. Their results showed that milk, cheese and CPP-ACP mousse were equally helpful in saturating saliva with adequate amounts of calcium and phosphate [34]. Two studies have shown that DP intake can modulate salivary microbiota and dental biofilm [35,36]. In particular, Johansson et al. found that low milk intake was associated with a higher prevalence of several opportunistic species, including caries-associated *S. mutans* [35]. 

**Table 1:** Characteristics and main findings of the included studies on milk and DPs.

Author, Year	Study Design	Population	Treatment/Test	Outcomes/Main Findings
Hegde et al., 2014 [34]	Case–control study	90 kindergarten children (5 yrs)	Milk and cheese	Salivary calcium and phosphate levels were higher than the baseline values after administration of milk, cheese and CPP-ACP mousse group at all intervals tested.
Zaki et al., 2015 [33]	Observational study	60 preschool children (2–6 yrs)	Relationship of dietary intake to ECC	Maintaining a healthy diet, particularly including DPs, has a significant protective effect against dental caries in preschool children.
Neves et al., 2016 [29]	Observational study	16 children (>24 mos)	Breast milk	Breastfeeding did not provoke a decrease in biofilm pH, irrespective of the children’s caries status.
Vakil et al., 2016 [37]	Review	Preschool children	Milk and DP	Information on the remineralization potential of milk and milk products is limited.
Nirunsittirat et al., 2016 [31]	Prospective Cohort Study	860 children (21 days–36 mos)	Breast milk	Breastfeeding up to 11 mos prevents dental caries in primary teeth. Prolonged breastfeeding was not associated with dental caries in the study population.
Johansson et al., 2018 [35]	Prospective Cohort Study	154 adolescents (17 yrs)	Self-reported total, non-fermented and fermented milk	Milk intake may modulate, but not prevent, the development of dental caries by reducing specific disease-associated bacterial species.
Branger et al., 2019 [30]	Review	Preschool children	Breast milk	Beyond the age of 1 year, it is difficult to determine whether breastfeeding can protect against or cause aggravation of caries because of a number of confounding factors (dietary and oral hygiene habits).
Sungkar et al., 2020 [36]	Observational study	22 adolescents (10–12 yrs)	Cheese and milk	Salivary buffer capacity after cheese consumption is higher than after milk consumption.
Sukmana et al., 2020 [28]	Review	Preschool children	Breast milk	It is important to educate parents, since the risk of caries in breastfed infants may increase after the first feeding of complementary foods.
Garcia-Pola et al., 2021 [38]	Observational study	166 children (6 yrs)	Milk and DP intake	It is critical to encourage protective behaviors such as fruit and milk consumption and minimize cariogenic foods considering the prevalence of caries observed in immigrant children.
Yardimci et al., 2021 [39]	Cross-Sectional Study	153 children (30–71 mos)	Milk and DP intake	Low-carbonated beverages, starchy food consumption, and high protein-containing beverages (milk and DPs), had a positive effect on dental health.
Wang et al., 2021 [32]	Observational study	6885 individuals (2–17 yrs)	Whole/low-fat/skim milk, yogurt, cheese, creams	High yogurt and low cheese intake are associated with a lower risk of dental caries among American children and adolescents.
Olczak-Kowalczyk et al., 2021 [40]	Observational study	1638 children (3 yrs)	Unsweetened milk	Limiting consumption of unsweetened beverages before bedtime can reduce the risk of caries.

Abbreviations: ECC: early childhood caries; DPs: dairy products; CPP-ACP: casein phosphopeptides and amorphous phosphate; yrs: years; mos: months.

#### 3.3.2. Milk and DPs Supplemented with PBs

Eighteen articles examined the effects of PB-supplemented DPs on caries development (Table 2). Five studies evaluated the effects on preschool children under 6 years of age. Piwat et al. and Sandoval et al. suggested that daily or three times weekly consumption of PB milk was sufficient to regress carious lesions and prevent new ones [41,42]. In addition, Xu et al. reported that the regular consumption induces changes in the structure and composition of the salivary microbiota. In particular, there is an increase in the *Campylobacter*, *Haemophilus*, *Lautropia*, *Bacillus*, *Catonella*, *Lactococcus*, and *Solibacillus* species and a decrease in *Gemella* and *Streptococcus* genera [43]. Additionally, Villavicencio et al. concluded that daily consumption of milk supplemented with *Lactobacillus rhamnosus* and *Bifidobacteruim longum* reduced the amount of *Lactobacillus* spp. and increased the buffering capacity of saliva [44]. Nine trials included children older than 6 years of age. In particular, studies of salivary microbial colonization reported a reduction in salivary *S. mutans* and, in general, CFU levels after PB consumption [45,46,47,48]. In contrast, other studies have suggested that consumption of PBs containing *Bifidobacterium lactis* does not reduce salivary levels of *S. mutans* and *Lactobacilli* [49] and has no effect on dental plaque [50]. Furthermore, Sakhare et al. concluded that PBs could have a short-term effect on salivary pH [22].

**Table 2:** Characteristics and main findings of included studies on milk and DPs supplemented with PBs.

Author, Year	Study Design	Population	Treatment/Test	Outcomes/Main Findings
Pinto et al., 2014 [51]	RCT	30 adolescents (15 yrs)	PB yogurt	The use of the tested probiotic strain (*B. animalis* subsp. *lactis*) for a period of 2 weeks provided no additional benefit.
Laleman et al., 2014 [52]	Review	Children (age not specified)	PB milk and DPs	PBs can have a positive effect on reducing the *S. mutans* counts as long as they are being used.
Caglar, 2014 [50]	RCT	52 children (8–10 yrs)	PB yogurt	*Bifidobacterium bifidum* DN-173 010 has no effect on the dental plaque.
Mahantesha et al., 2015 [48]	Pre–post study with no control group	50 children (6 and 12 yrs)	PB ice cream/PB drink	Probiotic organisms definitely have a role in reducing the salivary *S. mutans* level, and PB ice cream showed better results than PB drink.
Ashwin et al., 2015 [46]	RCT	60 children (6–12 yrs)	PB ice cream	PB ice cream containing *Bifidobacterium lactis* Bb-12 and *Lactobacillus acidophilus* La-5 can lead to a reduction in cariogenic organisms.
Lodi et al., 2015 [53]	RCT	10 children (age not specified)	PB fermented milk	PB fermented milk reduced the number of oral microorganisms.
Nozari et al., 2015 [49]	RCT	49 children (6–12 yrs)	PB yogurt	Daily PB yogurt containing *Bifidobacterium lactis* could not reduce salivary *S. mutans* and *Lactobacilli*, while normal yogurt could reduce the *S. mutans* significantly.
Villavicencio et al., 2018 [44]	RCT	363 preschool children (3–4 yrs)	PB milk	The daily consumption of milk supplemented with *Lactobacillus rhamnosus* and *Bifidobacteruim longum* reduces the *Lactobacillus* spp. quantity and increases the buffer capacity of saliva.
Alp and Baka, 2018 [54]	RCT	45 adolescents (12–17 yrs)	PB kefir	Daily kefir consumption decreased the salivary microbial colonization in orthodontic patients.
Patil et al., 2019 [47]	RCT	30 children (8–13 yrs)	PB milk	PB milk demonstrated efficacy comparable to that of F mouthwash in reducing salivary *S. mutans* counts and plaque scores.
Sivamaruthi et al., 2020 [55]	Review	Children (age not specified)	PB milk	The regular PB product consumption significantly reduced the risk of caries in children by inhibiting cariogenic bacteria and enriching commensal ones.
Piwat et al., 2020 [41]	RCT	487 children (37.6 ± 9.2 mos)	PB milk powder	PB milk intake either daily or three times weekly can modestly prevent new caries, but considerably reverse carious lesions.
Sandoval et al., 2021 [42]	RCT	42 children (2–3 yrs)	PB milk	Regular intake of PB-supplemented milk in preschool children with high caries risk decreased the occurrence of caries and the salivary levels of hβD-3.
Xu et al., 2021 [43]	Pre–post study with no control group	6 children (49.3 mos)	PB yogurt daily	The PB yogurt consumption alters the structure and composition of salivary microbiota.
Reddy et al., 2021 [45]	Interventional study	80 children (8–12 yrs)	Kefir milk, PB curd, PB drink	PB products like kefir milk and PB curd have shown an efficient role in the reduction of *S. mutans* compared to the control group.
Sakhare et al., 2021 [22]	RCT	62 children (6 and 12 yrs)	PB curd	PBs substitute decreases salivary *S. mutans* count after continuous consumption for 3 weeks, and there is no short-term effect on salivary pH.
Guru Vishnu et al., 2023 [56]	RCT	20 children (3–6 yrs)	PB yogurt	*S. mutans* levels and plaque pH both drastically decreased in the study group, while there was no difference in the control group.
Meng et al., 2023 [57]	Review	Preschool children	PB milk	PBs, particularly *Lactobacillus rhamnosus*, show potential in preventing dental caries, reducing the high concentration of *S. mutans* in saliva.

Abbreviations: RCT: randomized controlled trial; PB: probiotic; DPs: dairy products; F: fluoride; yrs: years; mos: months.

#### 3.3.3. Milk and DPs Supplemented with F

Five studies analyzed the addition of F to DPs (Table 3). Two of these [58,59] investigated the caries preventive effect of milk fluoridation on primary teeth at the community level. Kallagova et al. found that there was no significant effect of fluoridated milk consumption on oral hygiene indicators, salivation rate and oral fluid pH in children. However, regular consumption of fluoridated milk reduced the increase in caries in preschool children [58]. In line with these findings, Petersen et al. studied the supplementation of 0.5 mg F in 100 or 200 mL of milk or yogurt in two Bulgarian communities; the authors reported significantly less caries development than in schoolchildren receiving milk without added F [59]. Furthermore, Sköld-Larsson et al. suggested that daily consumption of fluoridated milk in adolescents undergoing orthodontic treatments may influence the balance between demineralization and remineralization of early enamel lesions [60].

Finally, Cagetti et al. and Yeung et al. conducted systematic reviews on the caries- preventive effect of fluoridated foods and showed that fluoridated milk has a beneficial effect on reducing the incidence and progression of caries in schoolchildren, but the scientific evidence remains weak [61,62]. 

**Table 3:** Characteristics and main findings of included studies on milk and DPs supplemented with F.

Author, Years	Design	Population	Treatment/Test	Outcomes/Main Findings
Sköld-Larsson et al., 2013 [60]	RCT	64 adolescents (13–18 yrs)	Fluoridated milk	Fluoridated milk can affect the equilibrium of de- and remineralization cycles of early enamel lesions adjacent to fixed orthodontic appliances.
Cagetti et al., 2013 [61]	Review	Children (3.5–4.5 yrs)	Fluoridated milk	The efficacy of milk fluoridation seems to be confirmed, but the availability of high-quality scientific data is poor.
Petersen et al., 2015 [59]	Cohort study	1498 children (3 yrs)	Fluoridated milk/yogurt	The use of fluoridated milk tested in Bulgarian schools indicates that this public health program can be effective in the overall fight against dental caries in children.
Yeung et al., 2015 [62]	Review	166 children (3 yrs)	Fluoridated milk	There is low-quality evidence to suggest fluoridated milk may be beneficial to schoolchildren, contributing to a substantial reduction in dental caries in primary teeth.
Kallagova et al., 2023 [58]	Observational study	2045 children (3–6 yrs)	Fluoridated milk	The milk fluoridation program is more effective for rarely and occasionally ill children with class 1 caries activity.

Abbreviations: RCT: randomized controlled trial; yrs: years.

### 3.4. Quality of the Articles Included

The risk of bias according to study design is shown in Table 4, Table 5, Table 6, Table 7 and Table 8. There was a moderate to high risk of bias in all study types assessed. In systematic reviews and meta-analyses (Table 4), the high risk was due to the criteria “focused question” and “bias assessed”. In observational cohort and cross-sectional studies (Table 5), the high risk was due to the criteria “study population”, “sample size”, “analyses”, “exposure(s) measures”, “blinding of outcome assessors”, and “confounding variables”. In case–control studies (Table 6), the high risk was due to the criterion “sample size”. In controlled intervention studies (Table 7), the high risk was due to the criteria “type of study”, “method of randomization”, “treatment allocation concealed”, “blinded information”, “blind evaluation”, “adherence to the intervention”, “sample size”, “outcomes reported/subgroups” and “analyze of randomized”. For pre–post studies without a control group (Table 8), the high risk was due to the criteria “study question”, “loss to follow-up” and “multiple outcome measures”.

## 4. Discussion 

This scoping review provides a broad overview of the evidence on the non-cariogenic effects of milk and DPs in children and adolescents, who are more susceptible to dental caries due to poor oral hygiene and unhealthy dietary habits [65,66]. 

### 4.1. Effect of Breast Milk 

During breastfeeding and childhood (up to the age of 6 years), factors such as poor sucking technique, nutrient intake, frequency of breastfeeding and the infant’s tooth structure may increase the risk of caries, leading to ECC and thus affecting oral health-related quality of life [28]. The literature reviewed agrees that breastfeeding up to 1 year does not increase the risk of dental caries because it does not significantly reduce the pH and may even be protective compared with formula feeding [29,30,31]. Moreover, consumption of sugary foods, especially drinks at bedtime, after 12 months of age may increase the risk of caries development [33,40]. Therefore, studies have reported that parental education on proper dental hygiene and dietary habits is a key factor in the prevention of ECC [39,40]. 

### 4.2. Effect of Milk and DPs 

Milk and DPs are important components of children’s diets. The majority of the studies analyzed demonstrated the beneficial and protective effects of milk and DPs on the incidence of dental caries. However, it should be noted that most of these studies were observational and were, therefore, more susceptible to bias and lack of randomization and may have had unpredictable results [67]. The benefits of milk and DPs can be attributed to several main factors: enamel remineralization, prevention of bacterial attachment to the teeth and inhibition of the ability of bacteria to form biofilms [12]. Milk constituents that may influence caries development include lactose, protein, fat, minerals and vitamins [12]. On the other hand, these components can prevent caries by competitively antagonizing enamel binding sites, improving the pH environment of plaque, inhibiting oral cariogenic bacteria, reducing enamel demineralization and promoting remineralization [37]. Recent articles suggest that levels of milk consumption are associated with distinct microbiota in saliva and dental biofilm [35,43]. Indeed, the development of dental caries is strongly associated with dysbiosis of the oral microbiota [53]. Studies are consistent in concluding that milk and cheese improve salivary saturation with essential calcium and phosphate and salivary buffering capacity [34,36].

One aspect to consider in the literature is the high frequency of consumption of beverages containing sucrose and added sugars. In fact, this habit increases plaque acidity and the potential for plaque formation and bacterial growth in the oral cavity [68].

### 4.3. Effect of Milk and DPs Supplements 

Many studies have investigated the association of milk and DPs with PBs and F to evaluate their anticariogenic effects. In particular, PBs have been shown to restore microbial populations associated with good oral health [69]. However, it is still unclear how PBs affect the composition and structure of the oral microbiota, particularly in children. Researchers have found that the use of PBs can reduce the number of pathogenic microorganisms associated with dental caries, providing a basis for predicting possible relationships between PB interventions and oral health in preschool children [43]. Some articles have described the beneficial effect of PBs, including buffering of salivary pH, production of bacteriocin and enzymes (dextranase, mutanase, and urease) and ability to compete for adhesion and colonization on tooth surfaces [55]. The currently available scientific literature on short-term follow-up of PB use is contradictory. For example, some studies supported that daily consumption of milk supplemented with *Lactobacillus rhamnosus* and *Bifidobacteruim longum* increased the buffering capacity of saliva in preschool children [44], and Piwat et al. suggested that daily or three times weekly consumption of PB milk can modestly prevent new caries and reverse carious lesions in young children [41]. Conversely, Pinto et al. and Caglar reported that the use of yogurt containing *Bifidobacterium bifidum* for 2 weeks was not sufficient to reduce the number of *S. mutans* and *lactobacilli* in the saliva or dental plaque of adolescent patients [50,51]. Nozari et al. confirmed that *Bifidobacterium lactis* had no inhibitory effect on *S. mutans* proliferation [49]. However, some studies concluded that PB substitute reduced salivary *S. mutans* counts after continuous use for 3 weeks, but had no short-term effect on salivary pH [22,48,57]. In addition, a recent review concluded that PBs reduce the *S. mutans* counts with a beneficial effect on caries prevention [52,55]. In general, the use of PB organisms to restore oral health and prevent caries is indeed gaining interest among researchers, although the mechanism of action of PBs is still unclear, as studies have only analyzed short-term effects (around 2 weeks). In fact, only two articles [42,43] have evaluated the long-term effect of PB-supplemented DPs in children over 1 year of age, concluding that regular consumption of PB-supplemented milk in preschool children at high risk of caries reduces the incidence of caries. However, there is a lack of relevant research, and further studies are needed to investigate the optimal dose of PB organisms and the long-term or synergetic effects of PB organisms on cariogenic bacteria and oral health. 

Few studies have been designed and conducted on the efficacy of F-supplemented milk and DPs [58,60]. F is a cornerstone in the prevention and arrest of dental caries due to its effects on enamel remineralization through fluorapatite formation, microbial metabolism and reduction of acid production by cariogenic bacteria [70]. The safety of F preparations in the correct concentrations used in modern dentistry is unquestionable, as confirmed by AAPD [71]. Therefore, F programs represent an important preventive strategy to reduce the risk of caries [72]. Our results showed that there is low-quality scientific evidence on the effectiveness of F supplementation in milk and DPs, but some studies showed a beneficial effect in school children in reducing caries incidence and progression. [61,62]. The lack of scientific interest in this topic may be due to the current availability of other F ingestion systems, such as water, toothpastes, or the use of topical products to prevent dental caries. 

Based on the data available in the scientific literature, this scoping review provides the clinician with an updated background on the non-cariogenic effect of milk and F in children and adolescents. Particularly now, that the clinical strategy for caries management is shifting from an invasive to a preventive approach, it is important to have a thorough understanding of the benefits of dietary components to oral health.

## 5. Limitations and Directions for Future Research

This scoping review has several limitations, even though it was conducted according to the PRISMA-ScR process. First, the three-database search strategy attempted to provide an accurate overview, but may not have identified all available sources, especially those in the grey literature. In addition, some references were excluded because they were not freely available. Second, despite the precautions and controls taken in the choice of keywords and the selection of articles, it is unreasonable to guarantee complete coverage. Third, there are differences between the included studies in terms of both study design and methodology. Indeed, the differences in materials and methods, as well as the specific aims of the different studies, are potential sources of bias and heterogeneity. Fourth, the included studies often used a non-homogeneous sample size, did not perform follow-up, and often did not describe how randomization and blinding were performed. In addition, there was considerable variation in study parameters, such as time of enrollment, type of application, study duration and outcome measures. Finally, another limitation may be the limited number of RCTs, which makes it difficult to confirm the effects of these products either alone or with the addition of PBs and Fs. Therefore, a future perspective for scientific research could focus on conducting rigorous scientific trials on the non-cariogenic effects of milk and DPs and their possible supplements, based on standardized scientific methodology with (i) longer follow-up periods, (ii) similar doses and durations of intake, (iii) comparable product types, (iv) larger and homogeneous samples, and (v) well-conducted and accurate analyses, in order to develop consistent and solid data and obtain high-quality and robust results. 

Finally, it may be interesting to delve deeper into this topic, using the presented scoping review as a starting point to identify a more specific thematic area using a limited search strategy for a future systematic review [73,74].

## 6. Conclusions 

This scoping review concluded that milk and DPs are generally beneficial and non-cariogenic in children and adolescents. The addition of PBs to DPs has been suggested to be beneficial and has shown the potential to influence the levels of cariogenic bacteria, such as *S. mutans* and *Lactobacillus* species, thereby providing a protective barrier against pathogenic microorganisms and supporting host defense. Furthermore, our results indicate that there is limited high-quality evidence to support the potential benefits of fluoridated milk. 

Therefore, taking into account the above limitations and considering that this was a scoping review [75,76], we could support the following recommendations for policy makers, clinicians and oral health professionals: (i) educate and motivate pregnant women to breastfeed until 1 year of age to prevent ECC; (ii) encourage the use of milk and DPs without added sugars; (iii) consider the use of milk and DPs supplemented by PBs in the diets of children and adolescents to promote the maintenance of an oral bacterial flora with low cariogenic potential. 

## Figures and Tables

**Table 4:** Risk of bias assessment for systematic reviews and meta-analyses using the NIH quality assessment tool. In the color-coded ranking, green color represents low risk of bias, orange some concerns, and red high risk of bias.

First Author, Year	Focused Question	Eligibility Criteria	Literature Search Strategy	Independent Review	Independent Rate	Characteristics and Results	Bias Assessed	Heterogeneity Assessed
Cagetti et al., 2013 [61]								
Laleman et al., 2014 [52]								
Yeung et al., 2015 [62]								
Vakil et al., 2016 [37]								
Branger et al., 2019 [30]								
Sukmana et al., 2020 [28]								
Sivamaruthi et al., 2020 [55]								
Meng et al., 2023 [57]								

**Table 5:** Risk of bias assessment for observational cohort and cross-sectional studies using the NIH quality assessment tool. In the color-coded ranking, green color represents low risk of bias, orange some concerns, and red high risk of bias.

First Author, Year	Research Question	Study Population	Participation Rate	Recruitment	Sample Size	Analyses	Timeframe	Exposures	Exposure Measures	Exposure(s) Measures	Outcome Measure	Blinding of Outcome Assessors	Loss to Follow-Up	Confounding Variables
Petersen et al., 2015 [59]														
Zaki et al., 2015 [33]														
Nirunsittirat et al., 2016 [31]														
Johansson et al., 2018 [35]														
Sungkar et al., 2020 [36]														
Wang et al., 2021 [32]														
Olczak-Kowalczyk et al., 2021 [40]														
Garcia-Pola et al., 2021 [38]														
Yardimci et al., 2021 [39]														
Kallagova et al., 2023 [58]														

**Table 6:** Risk of bias assessment for case–control studies using the NIH quality assessment tool. In the color-coded ranking, green color represents low risk of bias, orange some concerns, and red high risk of bias.

First Author, Year	Research Question	Study Population	Sample Size	Similar Population at Baseline	Eligibility Criteria	Cases-Controls	Randomization	Concurrent Controls	Exposure	Exposure Measures	Blinding of Outcome Assessors	Confounding Variables
Hegde et al., 2014 [34]												
Navit et al., 2020 [63]												

**Table 7:** Risk of bias assessment for controlled intervention studies using the NIH quality assessment tool. In the color-coded ranking, green color represents low risk of bias, orange some concerns, and red high risk of bias.

First Author, Year	Type of Study	Method of Randomization	Treatment Allocation Concealed	Blinded Information	Blind Evaluation	Similar Group at Baseline	Overall Drop-out Rate	Differential Drop-out Rate	Adherence to the Intervention	Other Interventions	Outcomes Assessment	Sample Size	Outcomes Reported/Subgroups	Analyze of Randomized
Sköld-Larsson et al., 2013 [60]														
Caglar, 2014 [50]														
Pinto et al., 2014 [51]														
Nozari et al., 2015 [49]														
Ashwin et al., 2015 [46]														
Lodi et al., 2015 [53]														
Neves et al., 2016 [29]														
Chi et al., 2016 [64]														
Alp and Baka 2018 [54]														
Villavicencio et al., 2018 [44]														
Patil et al., 2019 [47]														
Piwat et al., 2020 [41]														
Sakhare et al., 2021 [22]														
Sandoval et al., 2021 [42]														
Reddy et al., 2021 [45]														
Guru Vishnu et al., 2023 [56]														

**Table 8:** Risk of bias assessment for before–after (pre–post) studies with no control group using the NIH quality assessment tool. In the color-coded ranking, green color represents low risk of bias, orange some concerns, and red high risk of bias.

First Author, Year	Study Question	Eligibility Criteria	Study Population	Enrollment	Sample Size	Intervention	Outcome Measures	Blinding of Outcome Assessors	Loss to Follow-up	Pre-post Statistical Analysis	Multiple Outcome Measures	Group Level Intervention
Mahantesha et al., 2015 [48]												
Xu et al., 2021 [43]												

## Data Availability

The data presented in this study are available in article and supplementary material.

## Supplementary Materials

The following supporting information can be downloaded at: https://www.mdpi.com/article/10.3390/children11020149/s1, Table S1. Preferred Reporting Items for Systematic reviews and Meta-Analyses extension for Scoping Reviews (PRISMA-ScR) Checklist.
